# Towards a hybrid user interface for the visual exploration of large biomolecular networks using virtual reality

**DOI:** 10.1515/jib-2022-0034

**Published:** 2022-10-11

**Authors:** Michael Aichem, Karsten Klein, Tobias Czauderna, Dimitar Garkov, Jinxin Zhao, Jian Li, Falk Schreiber

**Affiliations:** Department of Computer and Information Science, University of Konstanz, Konstanz, Germany; Infection Program and Department of Microbiology, Biomedicine Discovery Institute, Monash University, Melbourne, Australia; Faculty of Information Technology, Monash University, Melbourne, Australia; Faculty of Applied Computer Sciences & Biosciences, University of Applied Sciences Mittweida, Mittweida, Germany

**Keywords:** hybrid user interfaces, immersive analytics, metabolic network models, systems biology, virtual reality

## Abstract

Biomolecular networks, including genome-scale metabolic models (GSMMs), assemble the knowledge regarding the biological processes that happen inside specific organisms in a way that allows for analysis, simulation, and exploration. With the increasing availability of genome annotations and the development of powerful reconstruction tools, biomolecular networks continue to grow ever larger. While visual exploration can facilitate the understanding of such networks, the network sizes represent a major challenge for current visualisation systems. Building on promising results from the area of immersive analytics, which among others deals with the potential of immersive visualisation for data analysis, we present a concept for a hybrid user interface that combines a classical desktop environment with a virtual reality environment for the visual exploration of large biomolecular networks and corresponding data. We present system requirements and design considerations, describe a resulting concept, an envisioned technical realisation, and a systems biology usage scenario. Finally, we discuss remaining challenges.

## 1 Introduction

Biomolecular networks model the relationships between biomolecules within specific organisms. One particular type of biomolecular networks are metabolic network models, especially including genome-scale metabolic models (GSMMs), which serve as knowledge bases that assemble the knowledge regarding the chemical reactions that happen inside organisms. With recent advances in high-throughput sequencing technologies and an increase in available genome annotations, model sizes and corresponding levels of detail are ever-growing. Since the first GSMM was reconstructed back in 1999 for *Haemophilus influenzae* [1], models for more than 6000 organisms [2] have been reconstructed, each covering the relationships of up to several thousand chemical reactions and respective metabolites. Associated research questions and applications span several areas of research, including biotechnology and pharmacology, where one example application is drug development. We mainly focus on metabolic network models in the further course of this paper. Most of our discussions however also directly apply to the more general case of biomolecular networks.

The most widely-used visualisation paradigm for metabolic models is to show a node-link diagram of the underlying network structure, where reactions and metabolites are represented using graphical shapes such as circles or rectangles, whereas relationships between them are represented using arrows (see Figure 3). Various corresponding tools and visualisation techniques have been proposed over the years [3], [4], [5], [6], [7], [8], [9], [10], [11], with many of them having found their way into the established tool stack of expert users. However, with the steady increase in size and complexity of these models, corresponding network visualisations often tend to become cluttered, and layout information is not necessarily part of the model itself. Hence, new methods for metabolic model visualisation are needed that reduce visual clutter, while still facilitating the understanding of mechanisms and underlying model architectures. One strategy to achieve this is to increase the interactivity and only provide details on demand. Another strategy is to increase the drawing space, either by using larger screens or display walls (2D/2.5D), or by drawing them in 3D altogether.

When it comes to 3D, the current availability of low-cost, high-performance immersive devices, such as virtual reality (VR) and augmented reality (AR) head-mounted displays (HMDs) or even the more expensive large (stereoscopic) display walls, has laid solid technological foundations for the young research area of immersive analytics (IA). Researchers in IA investigate *the use of engaging, embodied analysis tools to support data understanding and decision making* [12, p. 1]. Immersive analytics tools have been shown to be beneficial for several data types and application domains, e. g. for geospatial visualisations [13, 14] or visualisations of molecular interactions [15]. In virtual reality, networks can be easily represented in 3D, which was done in a series of studies, among other things with regard to navigation [16], perspectives [17], layout techniques [18], and preservation of the mental map [19].

There have also been early approaches for metabolic networks in 3D and VR environments [20], [21], [22], but despite their convincing beginnings, these approaches never seem to have become fully established. We have identified three potential reasons for this. First, these approaches were based on an earlier technology, which does not make direct application as easy as it is with current devices, such as VR/AR HMDs. Second, using virtual reality continuously for several hours, which is not unusual for the analysis of such models, comes with a couple of drawbacks, such as postural instability and motion sickness [23]. Third, within the specific application domain of metabolic modelling, there are several established standards, tools, and databases which analysts frequently incorporate in their analyses. However, these were not incorporated in previous approaches. While the first issue can be addressed by developing another system using the current technology, the second and third issue require to re-think the underlying approach. We therefore propose a hybrid user interface that combines a desktop environment (2D) with a virtual environment (3D), allowing for transitions between these two environments at any time during an analytic session. Such an interface allows to exploit and combine the specific advantages of both environments, and switching to the desktop environment every now and then ensures the individual VR sessions are not too long and at the same time the desktop environment provides an interface to familiar tools and established standards.

This paper is organised as follows. We begin with a brief review of related work in Section 2. In Section 3 we then discuss both requirements of a corresponding hybrid user interface, based on literature and discussions with domain experts, as well as general design considerations. Building on this, in Section 4 we describe the theoretical concept that we propose and explain an envisioned technical realisation in Section 5. In Section 6 we then demonstrate the applicability of our concept in the form of a usage scenario and finally discuss important remaining challenges in Section 7.

## 2 Related work

### 2.1 Visualisation of metabolic models

Methods for the visualisation of metabolic pathways and models have been studied for over 25 years now. On the one hand this includes early approaches for individual pathways, like the approach by Karp and Paley [3], where pathways are split up into circular, linear, and tree-like substructures, which are drawn individually, or the algorithm from Becker and Rojas [4], which combines circular, hierarchical, and force-directed drawing techniques accordingly. On the other hand, approaches have been presented for entire network models that contain several pathways. Metabopolis [5] for example uses a city map metaphor to draw large metabolic models.

Such drawings have also been investigated from a usability point of view. Bourqui and Purchase found that the incorporation of domain-specific drawing conventions into available automatic layouts does not reduce the user’s performance in related tasks [24]. Consequently, adjusting a drawing towards more familiar representation patterns does not necessarily have a negative impact on its overall efficiency.

The results from such works have then been assembled in a range of end-user tools, targeted at biochemists and bioinformaticians. These include CellDesigner [6], Cytoscape [25], Escher [7], iPath [9], LMME [11], Newt [10], and Pathway Tools [8]. For a comprehensive overview on corresponding methods and tools, the interested reader is referred to the recent work by Schreiber et al. [26].

To help analysts to quickly find their way in a visualisation of a metabolic model and to increase consistency across different graphical representations, the graphical standard SBGN (Systems Biology Graphical Notation) has been established [27]. SBGN is now more and more supported in corresponding tools [6, 10, 28].

### 2.2 Networks in virtual reality

With the recent availability of low-cost and high-performance immersive visualisation devices, such as VR and AR HMDs or large stereoscopic display walls, the visualisation of data in 3D has gained a lot of attention again [29]. Immersive analytics is a young research area, investigating the benefits of these technological advances and the role of immersion for data analysis and decision making. Also, in the field of network visualisation, where earlier works on drawings in three dimensions [30, 31] were followed by a certain scepticism, there are results now that provide evidence for the applicability of VR for the visual exploration of networks in 3D. These include traversal and exploration methods in VR [16], [17], [18], the impact on the mental map [19], and labelling in 3D [32].

Especially for metabolic networks and pathways, which are the subject of this work, several approaches have been proposed for the visualisation in 2.5D [33], 3D, and virtual reality. The system MetNetVR, which was proposed by Yang et al. [20], shows a 3D layout of a metabolic network in a CAVE [34] and offers additional information on a synchronised tablet. Rojdestvenski presented a system for the browser based visualisation of metabolic networks in 3D using circular layouts and allows to construct hierarchical overviews [21]. The approach by Qeli et al. allows to visualise a metabolic network in 3D and provides animated integration of related simulation data [22]. Kim et al. used HMD-based virtual reality to teach students the TCA cycle and found an increase in their ability to recall in comparison to traditional teaching methodology [35]. Finally, Sommer and Schreiber presented a hybrid system to visualise metabolic networks, consisting of a desktop setup and a fish tank VR monitor, in order to combine complex analytic workflows with meaningful spatial 3D visualisation [36]. For the area of systems biology in general, the potential of VR and AR technologies has been recently discussed by Turhan and Gümüş [37].

While showing the potential of VR for network analysis, most methods have not found their way into practical applications, quite possibly for some of the reasons discussed in Section 1. We therefore propose a hybrid user interface which can overcome some of the problems that previous approaches have faced.

### 2.3 Hybrid user interfaces and transitional interfaces

While virtual environments provide seemingly infinite space and a high level of immersion, required resolution and interaction precision are not yet on the same level as for established desktop systems. For instance, many newer HMD devices boast a high resolution, while incorporating other factors such as field of view, frame rate and pixel density, however, would require 4.724 Billion pixels per second [38] (1800 Hz refresh rate) to reach human visual acuity (not considering foveated rendering). For comparison, a typical 4 K consumer display with a refresh rate of 60 Hz requires around 498 Million pixels per second. In order to combine the individual strengths of different display environments, Feiner and Shamash introduced the concept of *hybrid user interfaces*, which they described as a *combination of devices that take advantage of the strong points of each* [39]. While Feiner and Shamash only considered the simultaneous combination of both devices, Hubenschmid et al. recently extended the definition to *asynchronous hybrid user interfaces* [40], where devices are not used simultaneously but sequentially, so that individual technologies can realise their full potential at a time.

When using such devices sequentially instead of simultaneously, one important question concerns the transition process between reality, augmented reality, and virtual reality [41]. The MagicBook [42] was an early concept of how such transitions could look like. Interfaces that allow for transitions between heterogeneous devices or environments represent a form of *transitional interfaces* [43], as defined by Grasset et al. In the light of cross reality hybrid systems, this concept has now once again received great attention [40].

Motivated by the mentioned benefits, the applicability of such hybrid user interfaces for specific applications and domain-specific workflows is currently explored by several researchers, e. g. for the analysis of X-ray data [44].

## 3 Requirements and design considerations for a hybrid user interface

### 3.1 Requirements

Since the first GSMM has been reconstructed, many applications of GSMMs have emerged [2, 45]. Ten years after the first reconstruction, Oberhardt et al. identified five major categories of applications: (O1) *contextualisation of high-throughput data*, (O2) *guidance of metabolic engineering*, (O3) *directing hypothesis-driven discovery*, (O4) *interrogation of multi-species relationships*, and (O5) *network property discovery* [45]. Another ten years later, Gu et al. identified six main applications: (G1) *production of chemicals and materials*, (G2) *drug targeting in pathogens*, (G3) *prediction of enzyme functions*, (G4) *pan-reactome analysis*, (G5) *modelling interactions among multiple cells or organisms*, and (G6) *understanding human diseases* [2].

While these high-level applications do not directly translate into requirements for a corresponding software system, there have been efforts to formalise the involved tasks from a visualisation perspective on a lower level [46, 47]. Saraiya et al. compiled a comprehensive set of 13 requirements for pathway visualisation systems. Through a follow-up evaluation of available tools, they identified the following five requirements as future research agenda: (S1) *pathway construction and update*, (S2) *information overlay*, (S3) *overlay data from high-throughput experiments*, (S4) *pathway overview and interconnectivity*, and (S5) *multi-scale pathways*. Murray et al. on the other hand have developed a task taxonomy for the analysis of biological pathway data that consists of three main categories. The first category, *attribute tasks*, is divided into (M1) *multivariate* (M2) *comparison*, (M3) *provenance*, and (M4) *uncertainty*. The second category, *relationship tasks*, is divided into (M5) *attributes*, (M6) *direction*, (M7) *grouping*, (M8) *causality*, and (M9) *feedback*. And the third category, *modification tasks*, is divided into (M10) *annotate* and (M11) *curate*.

Based on the above considerations as well as domain expertise of two of the authors of this paper, we selected the following core components for our proposed concept.

**Hierarchical interaction paradigm:** This component provides the possibility to visually explore hierarchical structures in a model. Hierarchical relations may either be predefined, such as assignments to classical pathways or cell compartments, or algorithmically determined, e.g. by graph clustering algorithms [48] or biologically motivated decomposition algorithms [49]. Hierarchies may also be recursive. Among others, this component supports tasks (S4), (S5), (M5), and (M7).

**Visual integration of related data:** This component provides the possibility to integrate data into the visual representation of a model, e. g. by mappings. Examples are resulting flux distributions from model simulations, measured transcriptomics data, and measured spatial distribution gradients. Among others, this component supports applications (O1) and (G2) and tasks (S3) and (M1).

**Comparison of related models:** This component provides the possibility to visually compare models that have a relation to each other. This includes different strains of the same organism or differently constrained versions of the same underlying model, where the latter may result from different drug treatments or different states of infections. Comparisons can involve two or more related models. Among others, this component supports applications (O4), (G2), (G4), and (G6), and task (M2).

In addition to the above core components, we identified a couple of aspects that experts are used to and which should be integrated into a hybrid workflow:

**Familiar graphical representations:** The integration of familiar graphical representations, such as established layouts, e. g. the KEGG [50] layouts, or graphical standards such as SBGN. We think that this component supports all of the above applications and tasks. In particular, requirement (S2) contains the definition of consistent representations for pathways and entities [46].

**Established databases:** The possibility to access established online databases, such as KEGG, Reactome [51], BioModels [52], or BIGG [53], during an analytic session in order to get additional verified information about a particular species, reaction or pathway. We think that this component supports all of the above applications and tasks. In particular, requirement (M3) contains the determination of studies that provide evidence for observed relationships [47].

**Familiar software:** The possibility to access and communicate with tools from the familiar software stack, including modelling, simulation, and analysis tools, during an analytic session. Because of this wide range of tools, we think that this component supports all of the above applications and tasks.

From a usability point of view, in addition to the traditional task performance, we focus on two other goals, which have previously been shown to be affected by immersive technologies. One of them is to increase memorability, which has been shown to improve using virtual reality [54], and in particular for metabolic pathways [35]. Ensuring high memorability is especially of interest in the context of distributed analytic sessions over time, where the long term process of model understanding and insight generation plays a major role. The second additional goal is to decrease the cognitive effort spent during analysis, which has also been studied for working with graph visualisations [55]. Especially with recent work showing that the use of VR might increase the cognitive effort experienced [56], this factor has to be considered carefully.

### 3.2 Design considerations

The first question when designing a hybrid user interface is which devices to incorporate. The possibilities range from standard desktop environments and handheld devices over (tracked) stereoscopic 3D displays and large monitor walls, up to AR and VR HMDs or even physical data representations. Obviously, each of these devices comes with its own pros and cons, which are still subject of ongoing research. We have decided for the combination of a desktop environment and a virtual reality HMD-based environment, as we on the one hand wanted to keep the desktop as familiar point of reference for the analysts and on the other hand based on the promising results for the visualisation of networks in HMD-based virtual reality that we discussed above.

Besides the general availability and suitability of specific devices, a primary design choice for a corresponding hybrid user interface is whether the involved devices are to be used synchronously, as proposed by Feiner and Shamash, or asynchronously (sequentially), as suggested by Hubenschmid et al. Synchronous usage in our case may include different parts of the virtuality continuum [41], such as rendering a desktop view within a virtual environment or rendering virtual holograms next to the real desktop. Asynchronous usage on the other hand may involve both environments independently and allow for transitions between them. We see a high potential for virtual reality in our case. At the same time, we would like to keep the desktop environment as familiar as possible and thus do not want to render it into the virtual environment. We therefore decided to use the asynchronous hybrid user interface paradigm as the base for our concept.

Another particular goal is the design of an appropriate transitional interface with support for the mental map of a user during transitions. The mental map itself has been studied for dynamic networks [57] and for networks in VR [19]. We consider preserving the mental map of a network during transitions as the key to a successful integration of the VR and desktop environments for this application case, because the more seamless the transitions are, the more likely they are to happen if beneficial, and the more likely each of the technologies can exploit its individual potential.

While keeping the mental map is usually associated with preserving characteristics of the representation, such as the encoding and layout, the design of these aspects needs to be investigated in order to find a good balance of preserving the visual impression across devices, which already might require adaptations such as colour adjustments, and of exploiting the strengths of the devices. A simple example is the use of the third dimension, introducing depth into the network layout. While the use of the additional dimension can greatly facilitate readability, e. g. by unfolding the network better, perceptual issues such as depth distortion as well as the influence of the perspective might either put a strain on the analyst or decrease perception quality of important network features. Similarly, while the choice of navigation and interaction metaphors and operations needs to be investigated for a consistent user experience across devices, requirements of the devices might either force adaptations or benefits from exploiting their affordances might outweigh disadvantages. In order to support orientation across the environments, the interface between external and internal representation, i. e. the mental map, needs to be consistent when switching between devices. The exploration of the design space for hybrid user interfaces requires further evidence from user studies to develop further insight into what works and what does not, and potentially to create guidelines that support system designers.

Closely related to the aspects of orientation, navigation, and mental map preservation is the question how to depict representative structures for traversal of the data and the surrounding context information. For example, a focus + context visualisation would show a subset of the data in a focus view, while representing the remainder as context [58], usually using abstractions or aggregations, i.e. with less detail and less screen space [59]. An important consideration here is the selection paradigm for the subsets in focus, which can be geometric, as it is for example the case for fisheye views [60] and spherical drawings [61], or logical [62], which has been shown to be beneficial for the case of metro maps by Wang and Chi [63] and in a usability study by Schaffer et al. [64]. For the case of metabolic models, subsystems or pathways contained in the model can serve as logical references and then be selected to be shown in the focus. Further design considerations here are whether there is one focal area or multiple foci [62], representation considerations such as the organisation of screen space and the encoding, as well as navigation in and interaction with the resulting views. An example for the latter is a hierarchical exploration approach that allows to drill down via a hierarchical structure, usually a tree, for which a variety of visualisation and interaction methods have been proposed [65, 66].

For the particular component of model comparison, an important design choice is whether networks are to be compared spatially separated into several views, and for example using a similar layout, or combined into a single view, which provides a better overview but may decrease the overall information contained due to data aggregation [67, 68]. From our discussions with experts and as the data we deal with is considered to be able to be aggregated in a meaningful way, we decided to use the combined view for our concept.

## 4 Proposed concept

**Figure 1:**
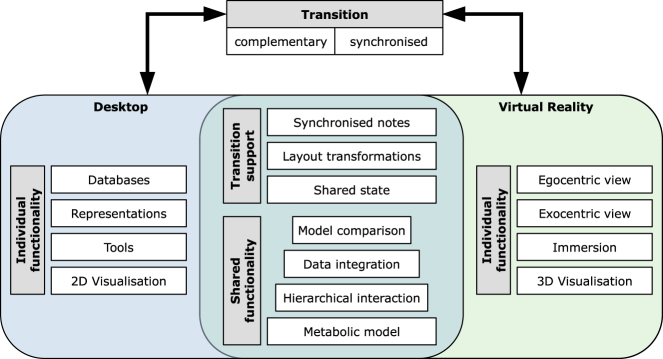
A schematic overview of the proposed concept for a hybrid user interface.

We propose a conceptual asynchronous hybrid user interface, combining a desktop environment with a virtual environment, that allows for transitions between the two environments at any time during an analytic session. While both of the environments provide individual features, there will be a base set of shared functionality (see Figure 1). We will first describe this shared functionality, which builds on our previous discussions and provides support for transitions, and then describe the individual features of each of the involved environments.

### 4.1 Shared functionality

The core of the system consists of loading metabolic models in different formats and visualising them as node-link diagrams, where the actual representation and layout are provided by the involved tools and depend on the environment. On top of this, we have the three basic components we identified, which were discussed above. They build upon each other to provide an analytic framework.

**Hierarchical interaction paradigm:** In order to reduce clutter and allow to explore hierarchical relationships, we follow a focus + context approach, where the focused parts are not determined geometrically but logically. One or more subsystems or pathways contained in the network model can be selected to be shown in the focus.

**Visual integration of related data:** Related data can be raw measured data, such as transcriptomics data from *in-vitro* experiments, as well as computationally derived data from *in-silico* experiments, such as flux distributions from a flux balance analysis (FBA) [69]. It is integrated into the model visualisation, using concepts like the mapping on visual variables (position, size, colour), according to the actual model representation in each of the two underlying environments. This data can then be explored using the hierarchical interaction paradigm, where visual integrations are sensitive to the focus and context state.

**Comparison of related models:** Related models include different strains of the same organism or differently constrained versions of the same underlying model, as they for example result from different drug treatments. The models can be shown as combined network, where structural differences and similarities are represented accordingly. For each model in comparison, data can be integrated, again resulting in a visual integration, combining the individual data, which can as before be explored using the hierarchical interaction paradigm.

### 4.2 Transitional interface

On top of the shared functionality, users are free to switch between the two environments at any time during an analytic session. Transitions can primarily be of two forms. First, the complementary usage of the different environments, caused by the switch to another subtask, for which the other environment is considered more appropriate. Second, the synchronised usage of the environments during one and the same subtask, in order to obtain a different perspective or change the interaction paradigm for the part of the data that is currently under investigation [40] (not to be confused with synchronous usage). We propose three main mechanisms to support analysts during transitions:

**Shared state:** The state of the analysis is synchronised across both environments to increase the consistency across transitions. The state in this case includes the metabolic model (individual or as combination of related models), the data that has been loaded and visually integrated into the model(s), together with the chosen mapping, and the current interaction state, which refers to the parts of the network that are focused and the parts that are contextualised.

**Synchronised notes:** In both involved environments, analysts have the possibility to add annotations and markings to elements and parts of the model, which has also been suggested by Hubenschmid et al. [40]. These notes are synchronised and help the analysts to link the two environments and to quickly find parts again after a transition that have been examined before the transition.

**Layout transformations:** Transitions are accompanied by layout transformations between 2D and 3D. By fixing one of the available dimensions in 3D, one can get a 2D projection of a layout. This can be used as an intermediate step between the 2D desktop and the 3D VR layout. Together with respective animations, this can help analysts to maintain their mental map [57].

### 4.3 Individual functionality

We now give a brief overview of the individual features for both environments.

The desktop environment is the current standard environment for most of the domain experts. It therefore serves as an interface to the established and familiar software components: biochemical databases and model repositories, such as KEGG, Reactome, BioModels, or BIGG, simulation, curation and analysis tools, such as CarveMe [70] or cobrapy [71], specific representations, such as SBML [72] or SBGN, and lastly the possibility to create figures for publications. Naturally, the starting point is in the desktop environment.

The VR environment, on the other hand, can provide a 3D layout of the underlying network structure, which provides more drawing opportunities, such as to reduce or avoid edge crossings, as well as some further arrangements of hierarchical structures in space. In addition, analysts can walk through their data (egocentric perspective), supporting spatial memory, or observe it from a distance (exocentric perspective) [17].

## 5 Envisioned technical realisation

**Figure 2:**
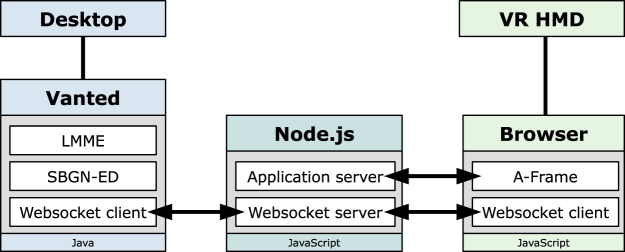
A schematic overview of the envisioned technical realisation of the proposed concept for a hybrid user interface.

The proposed concept is independent of an actual implementation. We will now provide an envisioned technical solution, which summarises our proof-of-concept implementation which is currently in development (see Figure 2). All components used in the implementation are open-source libraries and tools and we will also make our implementation available open-source.

### 5.1 Desktop environment

**Figure 3:**
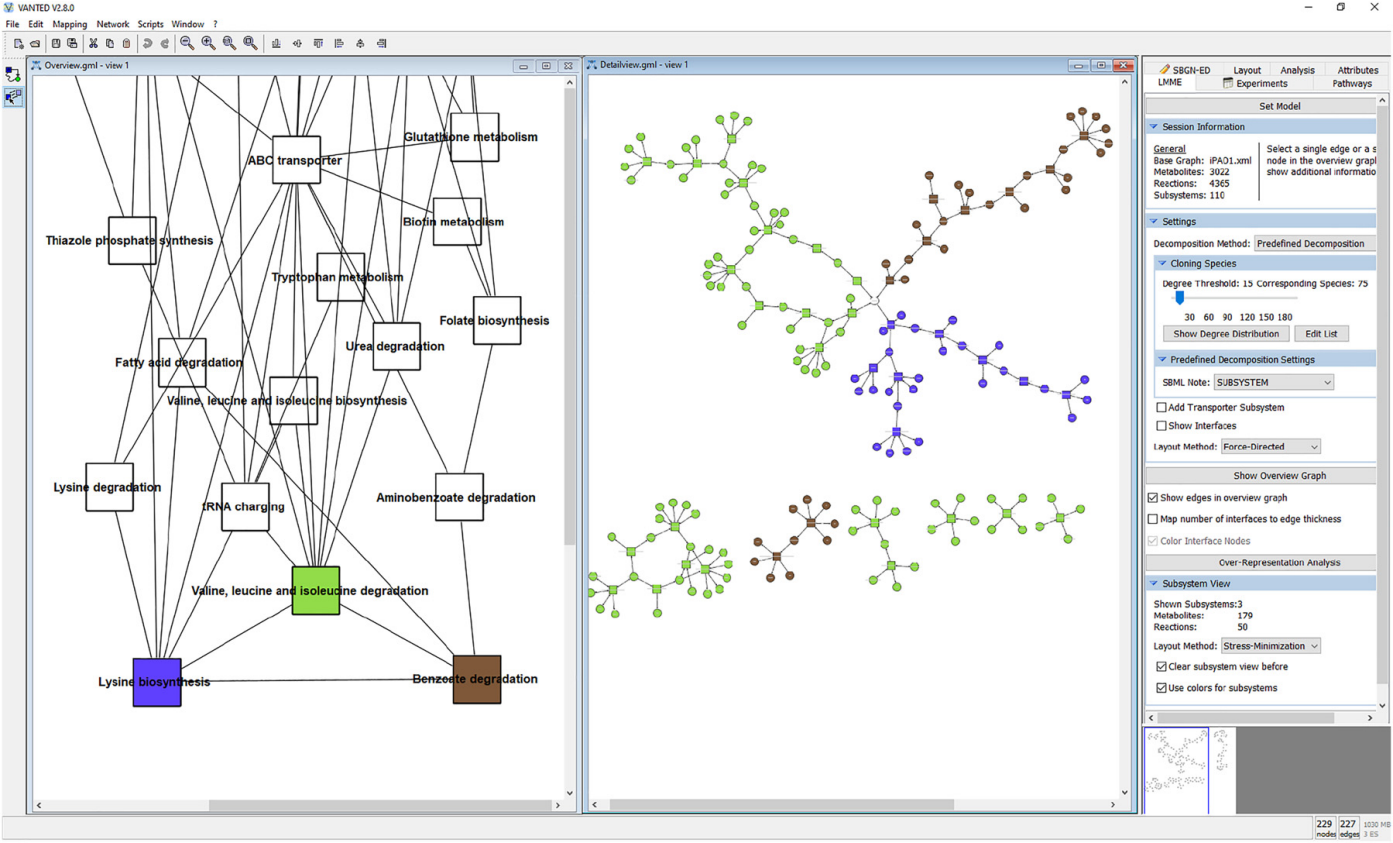
A 2D desktop exploration of *iPAO1*, a GSMM for *Pseudomonas aeruginosa* [79], using LMME. Three pathways have been selected in the overview (left) to be shown in detail (right): *Lysine biosynthesis* (blue), *Valine, leucine and isoleucine degradation* (green), and *Benzoate degradation* (brown).

For the desktop environment, we chose LMME (Large Metabolic Model Explorer) [11], a software for the hierarchical visual exploration of large metabolic models (see Figure 3). LMME itself is an add-on for Vanted [73], a tool for the visualisation and analysis of biological networks and related data. Vanted has built-in support for SBML and SBGN, the latter in particular through the SBGN-ED add-on [28]. In addition, Vanted provides access to several biological databases, including KEGG and BioModels. Vanted and LMME are both developed in Java.

### 5.2 Virtual environment

**Figure 4:**
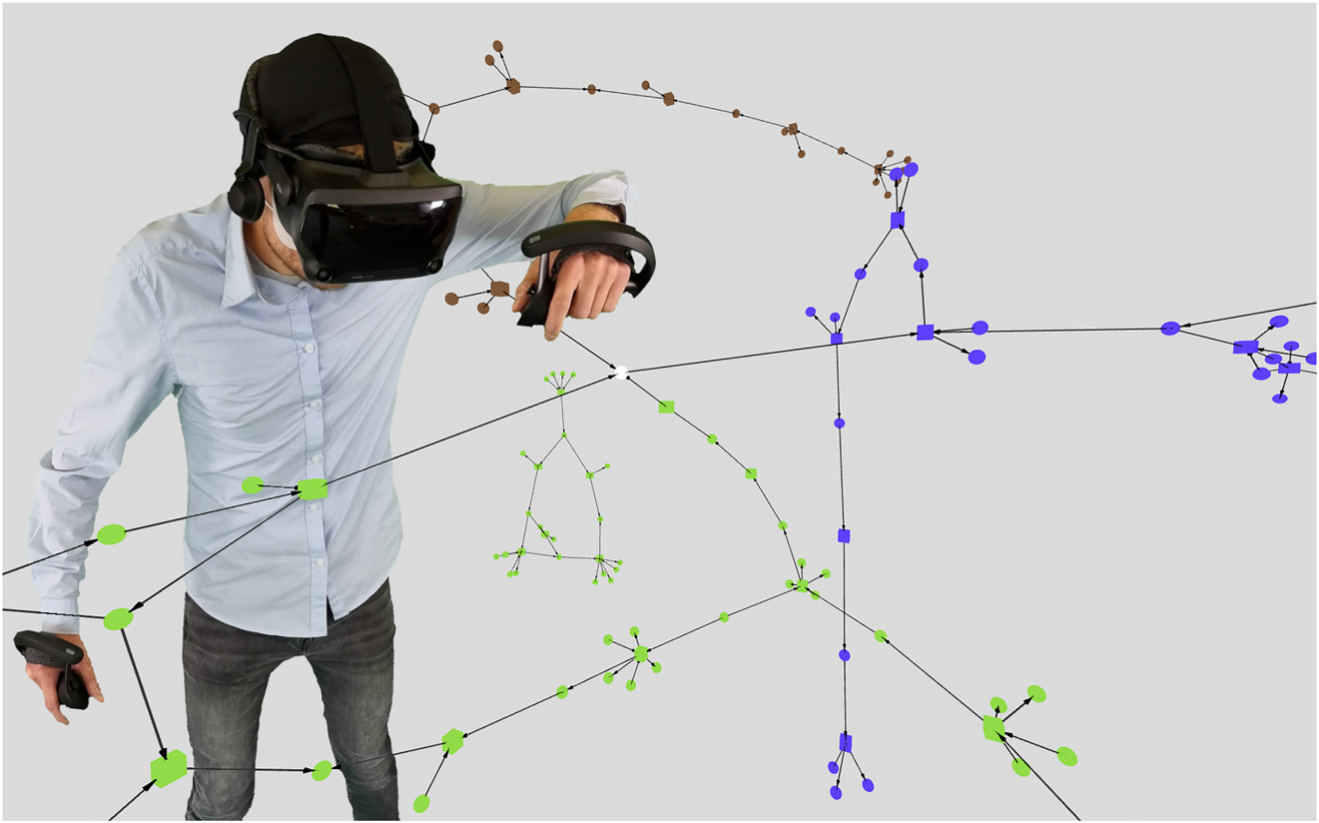
A 3D virtual reality representation using A-Frame. The same pathways are shown as in the detail view in Figure 3, using the same colour coding.

For the VR environment, we chose A-Frame [74], which is a web-based framework for the creation of VR and AR experiences in a web browser (see Figure 4, where we also used [75]). It is built on top of the powerful rendering-library three.js [76] and supports many of the current VR HMDs. For our VR environment, we will thus use JavaScript.

### 5.3 Connection

For the connection between both environments, we chose the WebSocket API [77], as it enables fast real-time bidirectional communication between clients. The WebSocket server is developed in JavaScript using the Node.js engine [78], while both environments contain a WebSocket client implementation in their respective programming languages.

We will evaluate our proof-of-concept implementation and further develop the transitional interfaces accordingly.

## 6 Usage scenario

Having described the system and its features, we would like to illustrate its usage in the form of a user scenario. Suppose we have a reconstructed metabolic model of a bacterial species. Using the resulting transcriptomics data from a drug treatment experiment, we were able to create two differently constrained model versions – one with treatment and one without. We now run two simulations using our preferred simulation tool and aggregate the resulting flux distributions into a single value per reaction. Using the desktop environment of our hybrid environment, we now load the model and map the flux data on the visualisation. We derive a decomposition of the model into several subsystems (or pathways) which we use as a base for our hierarchical exploration. We identify three subsystems that seem to have interesting flux patterns and select them for a detailed investigation. Due to their size, the detail view is still cluttered, such that we decide to switch to the virtual environment (synchronised usage). The state of the application is maintained such that we can quickly find our way in the new environment. While virtually walking through the selected subnetwork, we identify a series of chemical reactions that carry very different amounts of fluxes across the two model versions and therefore seem to play a key role in the overall mechanism. In order to find out more about the origin of this difference, we mark these reactions and go back to the desktop environment (complementary usage) and map the raw transcriptomics data for both model versions on the model in addition. Through this, we find that the reactions would indeed be able to carry fluxes in both versions and that there has to be another reason. We again switch to the virtual environment and inspect the wider neighbourhood of the reactions of interest. After some exploration, we indeed find that the series of reactions serves as a replacement for a reaction which has been inhibited by the drug treatment. We also mark this reaction and go back to the desktop environment. A targeted query of the marked reactions in an online database shows that a similar mechanism was found in another species. We correspondingly annotate the involved reactions and export the local subnetwork as a figure for later usage, e. g. using SBGN.

As we could see, there were three main reasons that initiated a transition. Switching from the desktop to the virtual environment happened because we wanted to inspect something in greater detail, while switching from the virtual to the desktop environment happened either because we wanted to perform another subtask which is provided by the latter or because we wanted to confirm a presumption that we made in the virtual environment. Similar reasons have also been discussed by Hubenschmid et al. [40].

## 7 Remaining challenges

The concept we proposed is a first step towards a hybrid user interface for large biomolecular network exploration. There are, however, many open questions and remaining challenges. We discuss some of them in the following.

### 7.1 Transition support

We described a couple of mechanisms to support users in their transition as part of our concept. There is, however, much work left in this regard. For example, instead of layout transformations between 2D and 3D, we could think about a more thorough investigation of the translation between a VR perspective, consisting of the network scaling, a three-dimensional position, and the viewing direction, on the one hand and the desktop perspective, consisting of a zoom level and a panning position, on the other hand. Moreover, methods are to be developed that assist analysts in their decision to switch between the involved environments – depending on the subtasks themselves as well as the personal preferences and habits. Supporting transitions around immersive environments has also recently been presented as one of the grand challenges in immersive analytics [80].

### 7.2 Evaluation

To verify the benefits of a hybrid interface, it has to be evaluated. One major goal in this case is to show that the benefits of combining the individual potentials of the used environments outweigh the additional cognitive effort that comes with the transitions. In addition, the preservation of the mental map of analysts through supporting methodology has to be quantified in order to design appropriate transitional interfaces. Especially this kind of evaluation has recently been discussed by Friedl et al. [81].

### 7.3 Collaboration

A key concept in immersive analytics is collaboration in immersive environments. In particular for the case of our hybrid interface, it has to be investigated how multiple analysts can make use of this collaboratively – from a conceptual as well as from a technical perspective. Questions arise such as how analysts can communicate during such sessions effectively, whether it is beneficial to have analysts using different involved environments simultaneously, and how we could construct scenarios for collaboration that allow analysts to diverge from and restore the shared state.

### 7.4 Involved environments

We have described a concept that combines a desktop environment and a VR environment. There are, however, more possible immersive environments and technologies that one could integrate, such as large display walls, 3D monitors, augmented reality (HMDs or mobile devices), and physicalisations of data. All of these have individually been shown to have great potential for data analysis. It is up to future research to explore the value of integrating such environments in addition, while maintaining smooth transitions.

## 8 Conclusions

We have presented a concept for a hybrid user interface for the visual exploration of large biomolecular networks, which combines a traditional desktop environment with a virtual reality environment. We discussed the requirements and design considerations of such a system and proposed a corresponding concept. In cooperation with domain experts, we have outlined a possible usage scenario, where we described the expected strengths of such a system. We currently work on a proof-of-concept implementation according to the envisioned technical realisation that we have provided, which will be made publicly available upon completion. Information about the current state can be found on https://cls.uni-konstanz.de/software/lmme/learn/vr-component. We plan to apply the system for a series of related investigations, targeting the general usability to facilitate the understanding of large biomolecular networks, the capability of different strategies to support transitions between environments, and the effects on memorability and cognitive effort needed. We think that immersive analytics research is in a state now, where respective approaches can demonstrate their potential to support domain-specific tasks and workflows, while the benefits outweigh the additional technological and cognitive efforts.
